# Common Genetic Variants and Risk for HPV Persistence and Progression to Cervical Cancer

**DOI:** 10.1371/journal.pone.0008667

**Published:** 2010-01-13

**Authors:** Sophia S. Wang, Paula Gonzalez, Kai Yu, Carolina Porras, Qizhai Li, Mahboobeh Safaeian, Ana Cecilia Rodriguez, Mark E. Sherman, Concepcion Bratti, Mark Schiffman, Sholom Wacholder, Robert D. Burk, Rolando Herrero, Stephen J. Chanock, Allan Hildesheim

**Affiliations:** 1 Division of Etiology, City of Hope National Medical Center, Duarte, California, United States of America; 2 Proyecto Epidemiológico Guanacaste, Fundación INCIENSA, San José, Costa Rica; 3 Division of Cancer Epidemiology and Genetics, National Cancer Institute, Bethesda, Maryland, United States of America; 4 Key Laboratory of Systems and Control, Academy of Mathematics and Systems Science, Chinese Academy of Sciences, Beijing, China; 5 Albert Einstein College of Medicine, Bronx, New York, United States of America; 6 Core Genotyping Facility, Division of Cancer Epidemiology and Genetics, Advanced Technology Program, SAIC Frederick Inc, NCI-Frederick, Frederick, Maryland, United States of America; Health Canada, Canada

## Abstract

HPV infrequently persists and progresses to cervical cancer. We examined host genetic factors hypothesized to play a role in determining which subset of individuals infected with oncogenic human papillomavirus (HPV) have persistent infection and further develop cervical pre-cancer/cancer compared to the majority of infected individuals who will clear infection.

We evaluated 7140 tag single nucleotide polymorphisms (SNPs) from 305 candidate genes hypothesized to be involved in DNA repair, viral infection and cell entry in 416 cervical intraepithelial neoplasia 3 (CIN3)/cancer cases, 356 HPV persistent women (median: 25 months), and 425 random controls (RC) from the 10,049 women Guanacaste Costa Rica Natural History study. We used logistic regression to compute odds ratios and p-trend for CIN3/cancer and HPV persistence in relation to SNP genotypes and haplotypes (adjusted for age). We obtained pathway and gene-level summary of associations by computing the adaptive combination of p-values. Genes/regions statistically significantly associated with CIN3/cancer included the viral infection and cell entry genes 2′,5′ oligoadenylate synthetase gene 3 (*OAS3*), sulfatase 1 (*SULF1*), and interferon gamma (*IFNG*); the DNA repair genes deoxyuridine triphosphate (*DUT*), dosage suppressor of mck 1 homolog (*DMC1*), and general transcription factor IIH, polypeptide 3 (*GTF2H4*); and the *EVER1* and *EVER2* genes (p<0.01). From each region, the single most significant SNPs associated with CIN3/cancer were *OAS3* rs12302655, *SULF1* rs4737999, *IFNG* rs11177074, *DUT* rs3784621, *DMC1* rs5757133, *GTF2H4* rs2894054, *EVER1/EVER2* rs9893818 (p-trends≤0.001). SNPs for *OAS3*, *SULF1*, *DUT*, and *GTF2H4* were associated with HPV persistence whereas *IFNG* and *EVER1/EVER2* SNPs were associated with progression to CIN3/cancer. We note that the associations observed were less than two-fold. We identified variations DNA repair and viral binding and cell entry genes associated with CIN3/cancer. Our results require replication but suggest that different genes may be responsible for modulating risk in the two critical transition steps important for cervical carcinogenesis: HPV persistence and disease progression.

## Introduction

Persistent infection with one of approximately 15 types of human papillomavirus (HPV) is necessary for the development of cervical cancer and its immediate precursor, cervical intraepithelial neoplasia grade 3 (CIN3). However, HPV infection is not a sufficient cause of cervical cancer/CIN3 and HPV cofactors have been identified, including oral contraceptive use and smoking [1]. Familial aggregation studies and evaluation of inherited genetic variations further suggest that host genetic factors may also contribute to cervical cancer pathogenesis [2]. A number of studies have evaluated and implicated a role for human leukocyte antigens (*HLA*) [3], [4]. We previously reported the association between common variants in genes influencing DNA damage, notably, *FANCA*, associated with both HPV persistence and progression to CIN3/cancer. We also reported a variant in the innate immunity gene *IRF3* associated with HPV persistence [5]. Here, we expand our evaluation of host genetic variations in DNA repair, viral infection and cell entry genes and risk for HPV persistence and cervical precancer/cancer.

We evaluated data on 7,140 candidate single nucleotide polymorphisms (SNP) in 305 candidate genes/regions (161 viral infection and cell entry and 144 DNA repair genes). All known DNA repair genes were included [6] and viral infection and cell entry genes were selected based on biological evidence of hypothesized association with cervical cancer, HPV, or other infections. All genes and their selected variants were evaluated for risk of HPV persistence and progression to CIN3/cancer within the population-based Guanacaste cohort in Costa Rica (genes and SNPs are annotated in Table S1). A unique aspect of our effort is the ability to separately evaluate genetic factors associated with the two known and critical transition states in the natural history of cervical cancer – (i) viral persistence and (ii) progression to pre-cancer/cancer.

## Results

### Pathway-Based Associations

We found the DNA repair pathway as a whole statistically significantly associated with CIN3/cancer (p = 0.0197) and with HPV persistence (p = 0.0472) but not progression (p = 0.7451) (Table 1). These associations appeared driven by genes involved in editing/processing nucleases, modulation of nucleotide pools, and nucleotide excision repair. The family of Fanconi anemia genes was also significantly associated with HPV persistence (p = 0.0326).

**Table 1:** Pathway-based tests for significance among DNA repair genes for associations with (i) cervical precancer/cancer, (ii) progression to cervical precancer/cancer, and (iii) HPV persistence.

DNA Repair Pathway	Genes	# genes	# SNPs	(i) CIN3/CA vs RC	(ii) CIN3/CA vs HPV persisters	(iii) HPV persisters vs RC
All genes		138	2985	**0.0197**	0.7451	**0.0473**
Base excision repair	*UNG, SMUG1, MBD4, TDG, OGG1, MUTYH, NTHL1, MPG, NEIL1, NEIL2, APEX1, APEX2, LIG3, XRCC1, PNKP, PARP1, PARP2*	17	363	0.1961	0.6703	0.1609
Chromatin Structure	*H2AFX, CHAF1A*	2	27	0.6365	0.0912	0.3588
DNA polymerases	*POLB, POLG, POLD1, POLE, PCNA, REV3L, MAD2L2, REV1L, POLH, POLI, POLQ, POLK, POLL, POLM, POLN*	14	289	0.2588	0.7481	0.6309
Direct reversal of damage	*MGMT, ALKBH3*	2	105	0.9515	0.9996	0.9975
Editing/processing nucleases	*FEN1, TREX1, TREX2, EXO1, SPO11, * ***FLJ35220***	6	95	**0.0096**	0.1961	0.3546
Genes defective in diseases associated with sensitivity to DNA damaging agents	*BLM, WRN, RECQL4, ATM*	4	101	0.2465	0.2051	0.0732
Fanconi anemia	*FANCA, FANCB, FANCC, FANCD2, FANCE, FANCF, FANCG, * ***FANCL*** *, FANCM, FANCN (PALB2), C19ORF40*	10	222	0.6062	0.2230	**0.0326**
Homologous recombination	*RAD51, RAD51C, RAD51L3, * ***DMC1*** *, XRCC2, XRCC3, RAD52, RAD54L, RAD54B, BRCA1, BRCA2, SHFM1, RAD50, MRE11A, MUS81, EME1, EME2*	17	364	0.1185	0.7500	0.2318
Mismatch excision repair	*MSH2, MSH3, MSH6, MSH4, MSH5, PMS1, MLH1, PMS2, MLH3, PMS2L3*	10	254	0.7061	0.1263	0.4253
Modulation of nucleotide pools	*NUDT1, * ***DUT*** *, RRM2B.*	3	51	**0.0181**	0.6242	0.2219
Non-homologous end-joining	*XRCC5, PRKDC, LIG4, XRCC4, DCLRE1C*	5	200	0.4068	0.7096	0.6697
Nucleotide excision repair	*XPC, RAD23B, CETN2, RAD23A, XPA, RPA1, RPA2, RAP3, ERCC2, ERCC3, GTF2H1, GTF2H2, GTF2H3, * ***GTF2H4*** *, GTF2H5, CDK7, CCNH, MNAT1, ERCC5, ERCC1, ERCC4, LIG1, ERCC6, XAB2, DDB1, DDB2, MMS19L*	26	607	**0.0438**	0.7382	0.2982
Other genes with suspected DNA repair function	*CDLRE1A, CDLRE1B, RPA4, APTX, NEIL3, RECQL, RECQL5, HEL308, RDM1*	9	139	0.1382	0.3793	0.2542
Other conserved DNA damage response genes	*ATR, RAD1, RAD9A, HUS1, RAD17, CHEK1, CHEK2, TP53, TREX1, PER1*	9	127	0.8380	0.8285	0.4497
Rad6 pathway	*UBE2A, UBE2B, RAD18, UBE2V2, UBE2N*	5	54	0.6558	0.9324	0.8099

P values are based on the adaptive rank truncated product method.

### Gene/Region-Based Associations

Table 2 shows results for 9 genes/regions associated with CIN3/cancer compared to random controls at p<0.01, ordered by their statistical significance. Six of the genes were considered notable with a FDR ≤0.2, including the DNA repair genes - *GTF2H4*, *DUT*, and *DMC1 –* and the viral infection and cell entry related genes - *OAS3, SULF1*, and *IFNG*. The association for *GTF2H4* and *SULF1* were also associated with HPV persistence (p = 0.005). Three regions were statistically significantly associated with progression to CIN3/cancer at p<0.05: *TMC6 (EVER1), TMC8 (EVER2)*, and *FLJ35220*. All gene-based results are shown in Table S2.

**Table 2:** Significance levels (p values) for (i) cervical precancer/cancer, (ii) progression to cervical precancer/cancer, and (iii) HPV persistence, among gene regions with p<0.01 for an association with cervical precancer/cancer.

Gene	Chromosome	# SNPs	(i) CIN3/CA	(ii) CIN3/CA	(iii) HPV persistence+
			vs. RC	vs. HPV persistence	vs. RC
*OAS3*	12q24.2	36	0.0013*	0.2537	0.1354
*GTF2H4*	6p21.3	15	0.0018*	0.5355	0.0054
*SULF1*	8q13.3	77	0.0030*	0.6935	0.0056
*DUT*	15q15-q21.1	14	0.0038*	0.2399	0.0612
*IFNG*	12q14	10	0.0038*	0.0511	0.9136
*DMC1*	17q25.1	14	0.0038*	0.5865	0.4742
*TMC8 (EVER2)*	17q25.3	22	0.0081	0.0287	0.8114
*TMC6 (EVER1)*	17q25.3	26	0.0098	0.0309	0.8457
*FLJ35220*	17q25.3	18	0.0094	0.0232	0.0864

* FDR p≤0.2.

Results for all gene-based tests are shown in Table S2.

### SNP-Based Associations

Consistent with our gene/region-based analyses, we found evidence of an altered risk (approximately 2-fold) for CIN3/cancer for one or more SNPs in eight of the nine genes/regions with p-trends ≤0.001 (Table 3, SNPs ordered alphabetically by gene name). Of the four genes associated with HPV persistence, SNPs in *DUT* (rs3784621), *GTF2H4* (rs2894054, rs6926723), *OAS3* (rs12302655), and *SULF1* (rs4737999, rs4284050, rs10108002) had significant p-trend<0.05. Of the genes/regions associated with progression to CIN3/cancer, SNPs in *IFNG* (rs11177074) and between *EVER2/TMC8* and *EVER1/TMC6* (rs9893818) were statistically significant at p<0.05. Odds ratios and 95% confidence intervals for these SNPs are shown in Table S3 (all data for all SNPs are shown in Table S4). We note that other SNPs associated with CIN3/cancer included those in the *OAS1*, *OAS2*, and *POLN* genes (Table 3). As for *OAS3*, the associations with *OAS1* and *OAS2* were significant for HPV persistence whereas *POLN* was associated with disease progression.

**Table 3:** Significance levels (p values) for (i) cervical precancer/cancer, (ii) progression to cervical precancer/cancer, and (iii) HPV persistence, among SNPs with p≤0.001 for an association with cervical precancer/cancer.

Gene	rs #	(i) CIN3/CA	(ii) CIN3/CA	(iii) HPV persistence
		vs. RC	vs. HPV persistence	Vs. RC
*DMC1*	RS5757133	0.0007	0.1590	0.1114
*DUT*	RS3784621	0.0004*	0.1280	0.0262
*GTF2H4*	RS2894054	0.0001*	0.8742	0.0002
*GTF2H4*	RS6926723	0.0004	0.6840	0.0034
*IFNG*	RS11177074	0.0003	0.0095	0.4011
*OAS1*	RS12307655	0.0005*	0.2790	0.0034
*OAS2*	RS718802	0.0001	0.1844	0.0057
*OAS3*	RS12302655	0.00008	0.1909	0.0025
*POLN*	RS17132382	0.0011	0.0103	0.5304
*SULF1*	RS4737999	0.0001*	0.8918	0.0020
*SULF1*	RS4284050	0.0007	0.4994	0.0020
*SULF1*	RS10108002	0.0010	0.5160	0.0181
*TMC8,TMC6*	RS9893818	0.0003	0.0019	0.8434

* p<0.05 in oncogenic HPV+ restricted analyses.

SNP p values with FDR p<0.2 are denoted with astericks. Complete results for SNP-based results are shown in Supplemental Materials (Table S3 (SNPs listed in Table 2) and Table S4 (all)).

### Haplotype Associations

Results from haplotype-based analyses (defined by blocks of linkage disequilibrium) were generally consistent with the gene/region- and SNP-based findings. Haplotypes statistically significantly associated with CIN3/cancer included SNPs implicated in SNP-based analysis (as shown in Table S5 for *DUT*, *GTF2H4* and *SULF1* where haplotype blocks could be constructed). No new regions of interest were identified in haplotype analysis using the sliding window approach of 3 SNPs (data not shown).

## Discussion

In this population-based study, we found nine genes/regions associated with CIN3/cancer, 6 of which remained significant at a FDR≤0.2 – three DNA repair genes (*GTF2H4*, *DUT*, and *DMC1*) and three viral infection and cell entry related genes (*OAS3, SULF1*, and *IFNG*). A unique aspect of our study is the ability to separately evaluate associations with the two important transition steps in the natural history of cervical cancer – viral persistence and progression to precancer/cancer. Of the regions/genes found to be important, the association was primarily with HPV persistence for *GTF2H4* and *SULF1*. *IFNG* and the epidermodysplasia verruciformis (EV)-associated genes (*TMC6-EVER1* and *TMC8-EVER2*) were primarily associated with progression to CIN3/cancer. All top genes derived from the gene/region- and SNP-based analyses are annotated and briefly described in Table 4.

**Table 4:** Description of top-ranked genes.

Gene (Alias)	Chromosome	Gene Description*	Gene ontology**	Additional Comments	Selected References
***DNA repair***					
*DMC1*	17q25.1 ?22q13.1	Dosage suppressor of mck1 homolog, meiosis-specific homologous recombination	Female gamete generation; meiosis; reciprocal meiotic recombination; spermatogenesis; ATP binding; DNA binding	Rad51 homolog, meiosis	(6)
*DUT*	15q15-q21.1	Deoxyuridine triphosphatase	DNA replication; nucleobase, nucleoside, nucleotide and nucleic acid metabolic process; dUTP disphosphatase activity	Modulation of nucleotide pools	(6)
*FLJ35220 (ENDOV)*	17q25.3	Hypothetical protein FLJ35220		Editing/processing nucleases; incision 3′ of hypoxanthine and uracil	(6)
*GTF2H4*	6p21.3	General transcription factor IIH, polypeptide 4	DNA repair; nucleotide-excision repair, DNA damage removal; RNA elongation from RNA polymerase II promoter	Nucleotide excision repair; core TFIIH subunit p52	(6)
*POLN (POL4P)*	4p16.2	Polymerase (DNA directed) nu	DNA-dependent DNA replication; DNA-directed DNA polymerase activity	DNA polymerase; DNA crosslink repair	(6)
***HPV binding/immune-related genes***					
*IFNG*	12q14	Interferon, gamma	Cell cycle arrest; cell motion; signal tranduction	Cytokine with role in innate/adaptive immunity against viral/bacterial infections; role in tumor control	OMIM
*OAS1*	12q24.2	2′-5′-oligoadenylate synthetase 1	Nucleobase, nucleoside, nucleotide and nucleic acid metabolic process	Role in resistance to viral infection via degradation of viral and cellular RNAs and impairment of viral replication. Also implicated in control of cell growth, differentiation, and apoptosis.	OMIM
*OAS2*	12q24.2	2′-5′-oligoadenylate synthetase 2	Nucleobase, nucleoside, nucleotide and nucleic acid metabolic process	Resistance to viral infection (see OAS1)	OMIM
*OAS3*	12q24.2	2′-5′-oligoadenylate synthetase 3	Nucleobase, nucleoside, nucleotide and nucleic acid metabolic process	Resistance to viral infection (see OAS1)	OMIM
*SULF1*	8q13.3	Sulfatase 1	Heparin sulfate proteoglycan metabolic process; arylsulfatase activity	Coreceptor for heparin-binding growth factors and cytokines; involved in cell signaling	OMIM
*TMC8 (EVER2)*	17q25.3	Transmembrane channel-like 8		Epidermodysplasia verruciformis susceptibility gene	(20)
*TMC6 (EVER1)*	17q25.3	Transmembrane channel-like 6		Epidermodysplasia verruciformis susceptibility gene	(20)

* As defined in NCBI Entrez Gene (http://www.ncbi.nlm.nih.gov/sites/entrez); **Gene Ontology annotation (http://www.geneontology.org/); OMIM: http://www.ncbi.nlm.nih.gov/sites/entrez; GeneCards: http://www.genecards.org; (1) Supplement to Wood RD, Mitchell M, Lindahl T Mutation Research 2005 (http://www.cgal.icnet.uk/DNA_Repair_Genes.html); (2) Orth G. Host defenses against human papillomaviruses: lessons from epidermodysplasia verruciformis. Curr Top Microbiol Immunol. 2008;321:59-83.

Results for our DNA repair pathway-based analyses were consistent with our individual SNP-based results. Specifically, we found that genes within nucleotide excision repair (which includes *GTF2H4*) and modulation of nucleotide pools (which includes *DUT*) to be associated with HPV persistence. The DNA repair family of editing/processing of nucleases was associated with progression to CIN3/cancer and though several genes were statistically significant, none were notable at FDR < 0.2. The association between the Fanconi Anemia family of genes with HPV persistence is also consistent with our previous analyses that reported *FANCA* variants associated with HPV persistence (5). There have been no reports of polymorphisms in *GTF2H4*, *DUT* or *DMC* with HPV persistence, cervical cancer, or any other cancer to date. However, *GTF2H4* is located on chromosome 6p21.3 within the *HLA* region and is thus of note as a number of studies have investigated *HLA* Class II and I genes with cervical cancer and have consistently identified alleles (e.g., *HLA*-DRB*1301) associated with cervical cancer [4]. Whether the observed association between *GTF2H4* variants and HPV persistence is due to its biological function and its capacity as a DNA repair gene or to potential linkage disequilibrium with *HLA* requires further evaluation.

Variants in two genes postulated to play a role in viral and HPV binding, *OAS3* and *SULF1*, were also associated with HPV persistence in our population. *OAS3* plays a role in resistance to viral infection via degradation of viral and cellular RNAs and impairment of viral replication. Specifically, the OAS family of genes (*OAS1, OAS2, OAS3*) is induced by interferon. When enzymatically active OAS are bound to viral RNA, RNase L is activated, resulting in the degradation of viral RNA [7]. Notably, *OAS1* and *OAS2* SNPs were also associated with HPV persistence in our population (Table 3). *SULF1* (sulfatase 1) is involved in cell signaling and is a coreceptor for heparin-binding growth factors and cytokines. Sulfs potentially play a role in a cellular feed-back mechanism where they edit the sulfation of multiple heparin sulfate proteoglycans [8]. This is of particular interest as heparin sulfate proteoglycans are thought to be the primary attachment factor for HPV and treatment with heparin and heparin sulfate have inhibited infection of some HPV types [9].

Three genes were associated primarily with progression to CIN3/cancer including *IFNG*, a cytokine that plays a role in innate immunity against viral or bacterial infections. It also induces the OAS family of genes which leads to degradation of viral RNA. We also report an association between genes in *EVER1/TMC6* and *EVER2/TMC8* and a SNP that lies between the two genes with progression to CIN3/cancer. Mutations in either *EVER1* or *EVER2* genes are well-documented in the rare skin carcinoma, epidermodysplasia verruciformis (EV), which is characterized by infection with HPV5 [10]. That common polymorphisms within *EVER1* and *EVER2* are also associated with progression to CIN3/cancer may potentially suggest a larger role, though modest, for *EVER1* and *EVER2* genes in HPV susceptibility and subsequent risk for disease.

As described previously [5], study limitations may include potential survival bias as the supplemental cases identified in Guanacaste were retrospectively ascertained and DNA was not obtained for deceased cases. Another limitation is our inability to evaluate invasive cervical cancer separately. Future studies with larger numbers of cases will be needed to address whether specific genes are involved in the transition from in situ to invasive disease. Although we targeted *a priori* genes and used a FDR of <0.2 to determine which genes were notable, we cannot exclude the possibility of false positives (or false negatives). Importantly, because our population comprises Costa Ricans and genes were tagged based on Caucasian and Yoruban populations where data are available from HapMap, it is plausible that gene coverage in our Costa Rican population is incomplete. Study strengths include our population-based study design. This design approximates a case-cohort design since the proportion of women in the cohort with CIN3 or cancer is small. Another study strength is our ability to evaluate genes relevant for HPV persistence and genes relevant for disease progression. Further, our study had rigorous follow-up, HPV testing and pathology review for case definitions. Our tag-SNP approach also allowed for broader coverage of each *a priori* gene/region investigation, compared to prior candidate SNP-based genotyping approaches. Our evaluation of the DNA repair pathway was near complete based on most recent literature and allowed for pathway-based analyses. However, our viral binding and cell entry genes were not amenable to similar analyses. We acknowledge that our *a priori* approach sampled a small subset of genes in the genome and we therefore likely missed other important associations. Increased power and replication of our results are required and larger efforts in genome-wide association studies should shed additional light on genes and regions of significance for cervical cancer. Our study, however, remains the only one designed to delineate between associations with HPV persistence and progression to CIN3/cancer.

In summary, our results require replication but build upon our previous report of potential host genetic variants relevant for HPV persistence and those relevant for progression to CIN3/cancer. If replicated, additional studies to pinpoint the causal SNP(s) and determine their biological relevance should be pursued. Future efforts should include further dissection of long-term persistence that likely leads to progression compared to short-term persistence that is less likely to progress to CIN3/cancer. The role of host gene and HPV methylation and their role on causal genes should also be considered. These data are important for future research in evaluating the interplay between viral and host genetics in determining risk of HPV persistence and for progression to CIN3/cancer.

## Methods

### Study Population

The present study was nested within a population-based cohort study of women in Guanacaste, Costa Rica. Details of the cohort study methods [11], [12] and of the sub-population selected for genetic analyses [5] have been reported elsewhere. Briefly, the Guanacaste HPV natural history study is a population-based cohort of 10,049 women recruited over an 18-month period in 1993–94 and followed for seven years. For cohort participants, cervical cells were available for HPV DNA testing as previously described [11], [12], and buffy coat specimens were available for host gene polymorphism testing.

Women selected for the genetic study included: (i) all women in the cohort histologically confirmed with prevalent or incident CIN3 or cancer (n = 184); (ii) all women in the cohort with evidence of HPV persistence, defined as women positive for the same HPV type at two consecutive visits (n = 432) (median length of persistence: 25 months); and (iii) a random selection of controls from the cohort (n = 492) [5]. As described previously [5], we also included 331 supplemental CIN3 and cancer cases from Guanacaste who were not participants in the natural history study but were independently diagnosed with CIN3 or cancer during the same period in which our natural history study was conducted. We note that these supplemental cases were slightly older because of the larger proportion of cancers compared to our cohort-based cases where there was a higher proportion of CIN3. Allele frequencies were comparable between our cohort and supplemental cases, justifying the combining of the two groups for genetic analyses (5).

### Ethics Statement

The study was approved by both the US NCI and Costa Rica Institutional Review Boards and all subjects signed informed consent.

### Laboratory Methods

#### DNA extraction

DNA was extracted from buffy coats with PureGene purification kits/Autopure protocol (Gentra Systems) at SeraCare (Frederick, MD). For the supplemental cases the DNA extraction was done at the University of Costa Rica using the same kit/protocol.

#### HPV testing

PCR-based HPV DNA testing with L1 MY09/MY11 consensus primer methods [11], [13], [14] was conducted on cervical cells stored in standard transport media (Qiagen, Germantown, MD) from the natural history study only. Because cervical cells were not obtained from the supplemental cases, HPV results are not available for these women. For purposes of HPV-restricted analyses, supplemental precancer/cancer cases are presumed to be HPV-positive due to the present knowledge that oncogenic HPV is an established and necessary risk factor for cervical precancer/cancer.

#### Host genotyping

Genotyping of tag SNPs from 305 candidate genes/regions (Genes and SNPs annotated in Table S1) hypothesized to be involved in cervical cancer progession or HPV persistence was conducted at the NCI Core Genotyping Facility (Advanced Technology Center, Gaithersburg, MD; http://snp500cancer.nci.nih.gov) [15] using a custom-designed iSelect Infinium assay (Illumina, www.illumina.com). The Infinium included a total of 27,904 tag SNPs, of which our candidate genes/regions represented 7,765 SNPs. Tag SNPs for the 305 candidate genes were chosen from the designable set of common SNPs (minor allele frequency (MAF)>5%) genotyped in the Caucasian (CEU) and Yoruban (YRI) population sample of the HapMap Project (Data Release 20/Phase II, NCBI Build 36.1 assembly, dbSNPb126) using the software Tagzilla (http://tagzilla.nci.nih.gov/), which implements a tagging algorithm based on the pairwise binning method of Carlson *et al*. [16]. Because there is no Costa Rican population in the HapMap Project for which we could select SNPs, we included tagging for the YRI in addition to the CEU population to improve our probability of achieving gene coverage in our population. For each original target gene, SNPs within the region spanning 20 kb 5′ of the start of transcription (exon 1) to 10 kb 3′ of the end of the last exon were grouped using a binning threshold of r^2^>0.8 to define a gene/region. When there were multiple transcripts available for genes, only the primary transcript was assessed.

#### Quality control (QC)

Tag SNPs that failed manufacturing (ordered but did not convert), failed validation (no amplification or clustering) and assays that had less than 80% completion or 80% concordance with the 90 Hapmap CEU samples used for validation were excluded (n = 104). SNPs with low completion rate (<90% of samples) were further excluded (N = 138). SNPs with QC discordance among our 100 QC duplicates and among HapMap samples <98% were excluded (n = 383). We also excluded samples with a low completion rate (<90%) (N = 7). Hardy–Weinberg equilibrium was evaluated among controls. SNPs showing evidence of deviation from Hardy–Weinberg proportions (n = 49, p<0.0001) are denoted in Table S1. Though our QC data did not suggest any obvious genotyping error and we present their results, we note caution in interpretation of these select results. Of the 7,765 a priori SNPs, 7,140 SNPs were included in our present analysis.

#### Final analytic population

We evaluated a total of 416 women diagnosed with CIN3 or cancer, 356 women with HPV persistent infection, and 425 random controls for whom validated genotyping results were obtained.

### Statistical Analysis

#### Pathway- and gene/region-based analyses

We obtained pathway- and gene/region-based summary of associations using the adaptive combination of p-values [17], [18], which combines gene-level association evidence through adaptive rank truncated product method. To account for multiple comparisons, we applied the false discovery rate (FDR) method of Benjamini and Hochberg [19]. Pathway-based analyses were conducted for DNA repair genes; they were not conducted for viral infection and cell entry genes as only select genes were targeted for evaluation and together do not fully represent a pathway.

#### SNP-based associations

We calculated odds ratios (OR) and 95% confidence intervals (95% CI) for each genotype with each disease outcome, using the homozygous wild type (WT) genotype as the referent group. We first compared CIN3/cancer cases to random controls. We further evaluated whether the statistically significant associations for CIN3/cancer were consistent for HPV persistence and/or disease progression with the following respective comparisons: (i) HPV persisters compared to random controls and (ii) CIN3/cancer cases compared to HPV persisters.

We conducted both crude and analyses adjusted for age (<30, 30–49, 50+ years). For each outcome, we calculated the *P*_trend_ based on the three-level ordinal variable (0, 1, and 2) of homozygote WT, heterozygote, and homozygote variant in a logistic regression model. For evaluating associations with HPV persistence, we also conducted analyses where HPV persistence and controls were restricted to any oncogenic-HPV infection (16, 18, 31, 33, 35, 39, 45, 51, 52, 56, 58, 59, 68). All logistic regression models were unconditional and conducted using SAS version 9.1 (SAS Institute, Cary, NC).

#### Haplotype analyses

We conducted haplotype analyses using two methods. First, we evaluated risk of cancer, progression and HPV persistence associated with haplotypes defined by SNPs within a sliding window of three loci across a gene (Haplo Stats, version 1.2.1, haplo.score.slide, http://mayoresearch.mayo.edu/mayo/research/schaid_lab/software.cfm) on all genes. A global score statistic was used to summarize the evidence of association of disease with the haplotypes for each window. Second, we visualized haplotype structures for genes where p<0.01 for gene/region-based analysis using Haploview, version 3.11 [20] based on measures of pairwise linkage disequilibrium between SNPs. For blocks of linkage disequilibrium, we obtained ORs and 95% CIs for the underlying haplotypes under the assumption of an additive model (haplo.glm, minimum haplotype frequency 1%). All haplotype analyses were adjusted for age.

## Acknowledgments

We sincerely thank Drs. Chris Buck and Patricia Day for their scientific contributions in suggesting candidate genes for evaluation with regard to their relevance in HPV binding. We are grateful to Sabrina Chen from the Information Management Services, Inc. (IMS) for data management and programming support. We are grateful to Amy Hutchinson and Belynda Hicks for their management of this genotyping effort at the NCI Core Genotyping Facility and for their scientific contributions to our research efforts. Results were presented in part at the 25^th^ International Papillomavirus Conference, Malmo, Sweden, May 2009.

## Supporting Information

Table S1 (0.83 MB XLS)

Table S2 (0.61 MB DOC)

Table S3 (0.11 MB DOC)

Table S4 (5.99 MB XLS)

Table S5 (0.09 MB DOC)
