# Supplementary material for: Common Genetic Variants and Risk for HPV Persistence and Progression to Cervical Cancer
Source: PLoS One. 2010 Jan 13;5(1):e8667. doi: 10.1371/journal.pone.0008667 (PMC2801608; doi:10.1371/journal.pone.0008667)
Supplement: Table S2 — (0.61 MB DOC) [file pone.0008667.s002.doc]

**Table S2**. All results for gene-based tests for associations with (i) cervical precancer/cancer, (ii) progression to cervical precancer/cancer, and (iii) HPV persistence.

|  | SNPs | (i)  CIN3/cancer  vs RC |  | (ii)  CIN3/cancer  vs HPV persistence |  | (iii)  HPV persistence  vs RC |
| --- | --- | --- | --- | --- | --- | --- |
| **Gene** | **(#)** | **p** |  | **p** |  | **p** |
| ALKBH2 | 19 | 0.0323 |  | 0.2864 |  | 0.5495 |
| ALKBH3 | 31 | 0.8869 |  | 0.9914 |  | 0.9424 |
| APEX1 | 38 | 0.1408 |  | 0.7524 |  | 0.0161 |
| APEX2 | 8 | 0.2339 |  | 0.2574 |  | 0.6117 |
| APTX | 9 | 0.6062 |  | 0.7223 |  | 0.9836 |
| ATM | 36 | 0.2236 |  | 0.1786 |  | 0.9744 |
| ATR | 19 | 0.4472 |  | 0.6673 |  | 0.5159 |
| B3GAT3 | 7 | 0.7963 |  | 0.4201 |  | 0.2317 |
| B4GALT7 | 10 | 0.3991 |  | 0.1902 |  | 0.6860 |
| BAK1 | 34 | 0.5269 |  | 0.9498 |  | 0.8306 |
| BIRC5 | 17 | 0.8011 |  | 0.7715 |  | 0.3079 |
| BLM | 29 | 0.2964 |  | 0.7160 |  | 0.0858 |
| BRCA1 | 14 | 0.6362 |  | 0.0863 |  | 0.0180 |
| BRCA2 | 56 | 0.9359 |  | 0.5141 |  | 0.8219 |
| BRD4 | 12 | 0.5294 |  | 0.7309 |  | 0.2530 |
| BRIP1 | 18 | 0.5196 |  | 0.5425 |  | 0.7813 |
| C15orf48 | 13 | 0.2127 |  | 0.1598 |  | 0.0632 |
| C19orf40 | 19 | 0.1029 |  | 0.7633 |  | 0.1156 |
| CADM1 | 51 | 0.4124 |  | 0.0250 |  | 0.3213 |
| CAMP | 3 | 0.4724 |  | 0.5409 |  | 0.2222 |
| CASP10 | 17 | 0.4762 |  | 0.1634 |  | 0.5514 |
| CASP2 | 8 | 0.9680 |  | 0.1203 |  | 0.5488 |
| CASP3 | 21 | 0.2687 |  | 0.5232 |  | 0.1482 |
| CASP6 | 22 | 0.6186 |  | 0.7430 |  | 0.1289 |
| CASP7 | 42 | 0.9700 |  | 0.6003 |  | 0.7317 |
| CASP8 | 33 | 0.0741 |  | 0.1032 |  | 0.2638 |
| CASP9 | 17 | 0.0249 |  | 0.3183 |  | 0.1651 |
| CCND1 | 17 | 0.4026 |  | 0.3381 |  | 0.3440 |
| CCNH | 14 | 0.5695 |  | 0.5934 |  | 0.8365 |
| CD83 | 40 | 0.2270 |  | 0.0308 |  | 0.4428 |
| CDH1 | 57 | 0.1278 |  | 0.9350 |  | 0.5800 |
| CDK7 | 13 | 0.9475 |  | 0.9920 |  | 0.8667 |
| CDKN1A | 22 | 0.8705 |  | 0.5364 |  | 0.7783 |
| CDKN2A | 35 | 0.5766 |  | 0.0765 |  | 0.5426 |
| CDKN2B | 22 | 0.4110 |  | 0.0441 |  | 0.3618 |
| CETN2 | 15 | 0.8418 |  | 0.8958 |  | 0.3491 |
| CHAF1A | 10 | 0.5265 |  | 0.1444 |  | 0.4096 |
| CHEK1 | 12 | 0.3171 |  | 0.6920 |  | 0.7496 |
| CHEK2 | 23 | 0.2903 |  | 0.0794 |  | 0.4588 |
| CTLA4 | 19 | 0.9620 |  | 0.2864 |  | 0.5495 |
| CYBA | 12 | 0.4324 |  | 0.9914 |  | 0.9424 |

***Table S2 (continued)***

|  | SNPs | (i)  CIN3/cancer  vs RC |  | (ii)  CIN3/cancer  vs HPV persistence |  | (iii)  HPV persistence  vs RC |
| --- | --- | --- | --- | --- | --- | --- |
| Gene | (#) | p |  | p |  | p |
| DCLRE1A | 8 | 0.0709 |  | 0.0329 |  | 0.7158 |
| DCLRE1B | 11 | 0.3010 |  | 0.1353 |  | 0.3988 |
| DCLRE1C | 18 | 0.5527 |  | 0.8836 |  | 0.5690 |
| DDB1 | 8 | 0.8577 |  | 0.9756 |  | 0.9430 |
| DDB2 | 21 | 0.7414 |  | 0.3971 |  | 0.3073 |
| DEFA1 | 8 | 0.9247 |  | 0.4558 |  | 0.5693 |
| DEFA3 | 17 | 0.1385 |  | 0.0928 |  | 0.1024 |
| DEFA4 | 41 | 0.9329 |  | 0.6344 |  | 0.3434 |
| DEFA5 | 19 | 0.2444 |  | 0.3407 |  | 0.7334 |
| DEFA6 | 43 | 0.9364 |  | 0.5865 |  | 0.4742 |
| DMC1 | 14 | 0.0031 |  | 0.7053 |  | 0.8203 |
| DNAJC3 | 18 | 0.6335 |  | 0.2399 |  | 0.0612 |
| DUT | 14 | 0.0026 |  | 0.9774 |  | 0.9898 |
| EGF | 91 | 0.9668 |  | 0.4546 |  | 0.4900 |
| EIF2AK2 | 29 | 0.6232 |  | 0.1909 |  | 0.1263 |
| EME1 | 19 | 0.1137 |  | 0.5644 |  | 0.5681 |
| EME2 | 13 | 0.4698 |  | 0.1652 |  | 0.3889 |
| ERCC1 | 14 | 0.5825 |  | 0.6769 |  | 0.8244 |
| ERCC2 | 15 | 0.9478 |  | 0.1152 |  | 0.3212 |
| ERCC3 | 22 | 0.3795 |  | 0.9175 |  | 0.2197 |
| ERCC4 | 37 | 0.0433 |  | 0.7054 |  | 0.8166 |
| ERCC5 | 20 | 0.5803 |  | 0.5431 |  | 0.9960 |
| ERCC6 | 40 | 0.0458 |  | 0.1398 |  | 0.9283 |
| ERCC8 | 17 | 0.7244 |  | 0.5564 |  | 0.9574 |
| ESR1 | 86 | 0.4800 |  | 0.0541 |  | 0.2547 |
| ESR2 | 36 | 0.3032 |  | 0.7463 |  | 0.3437 |
| EXO1 | 25 | 0.0748 |  | 0.7081 |  | 0.0134 |
| FAF1 | 65 | 0.0982 |  | 0.7830 |  | 0.2121 |
| FANCA | 42 | 0.2012 |  | 0.7218 |  | 0.1681 |
| FANCB | 7 | 0.6738 |  | 0.7423 |  | 0.1464 |
| FANCC | 33 | 0.3193 |  | 0.2536 |  | 0.6532 |
| FANCD2 | 11 | 0.6289 |  | 0.0201 |  | 0.2383 |
| FANCE | 23 | 0.2114 |  | 0.9660 |  | 0.1402 |
| FANCF | 12 | 0.3736 |  | 0.9561 |  | 0.7905 |
| FANCG | 15 | 0.6047 |  | 0.6555 |  | 0.4471 |
| FANCL | 42 | 0.1011 |  | 0.4758 |  | 0.8378 |
| FANCM | 16 | 0.2052 |  | 0.1377 |  | 0.6567 |
| FAS | 29 | 0.5184 |  | 0.5195 |  | 0.6220 |
| FASLG | 17 | 0.9303 |  | 0.0329 |  | 0.7158 |
| FCGR2A | 13 | 0.2807 |  | 0.1353 |  | 0.3988 |
| FCGR3A | 13 | 0.4523 |  | 0.8836 |  | 0.5690 |
| FEN1 | 12 | 0.3049 |  | 0.9756 |  | 0.9430 |

***Table S2 (continued)***

|  | SNPs | (i)  CIN3/cancer  vs RC |  | (ii)  CIN3/cancer  vs HPV persistence |  | (iii)  HPV persistence  vs RC |
| --- | --- | --- | --- | --- | --- | --- |
| Gene | (#) | p |  | p |  | p |
| FLJ35220 | 18 | 0.0104 |  | 0.0232 |  | 0.0864 |
| FURIN | 14 | 0.2357 |  | 0.9620 |  | 0.6532 |
| GNS | 16 | 0.6105 |  | 0.9537 |  | 0.7077 |
| GPC1 | 23 | 0.0572 |  | 0.1620 |  | 0.7416 |
| GPC3 | 49 | 0.8683 |  | 0.9240 |  | 0.8915 |
| GPC4 | 18 | 0.1973 |  | 0.1324 |  | 0.9359 |
| GTF2H1 | 25 | 0.4048 |  | 0.2445 |  | 0.8693 |
| GTF2H3 | 25 | 0.9694 |  | 0.9891 |  | 0.9277 |
| GTF2H4 | 15 | 0.0007 |  | 0.5355 |  | 0.0054 |
| GTF2H5 | 10 | 0.0720 |  | 0.6687 |  | 0.2160 |
| H2AFX | 17 | 0.4342 |  | 0.3024 |  | 0.2634 |
| HEL308 | 9 | 0.5776 |  | 0.4297 |  | 0.1508 |
| HIF1A | 26 | 0.4273 |  | 0.8233 |  | 0.4336 |
| HS2ST1 | 33 | 0.2188 |  | 0.1796 |  | 0.7075 |
| HS3ST1 | 57 | 0.2660 |  | 0.2804 |  | 0.6812 |
| HS3ST2 | 89 | 0.0714 |  | 0.2626 |  | 0.3686 |
| HS6ST1 | 50 | 0.0719 |  | 0.4533 |  | 0.1381 |
| HS6ST2 | 31 | 0.2386 |  | 0.5960 |  | 0.2779 |
| HSP90AA1 | 18 | 0.0989 |  | 0.4665 |  | 0.1159 |
| HSPG2 | 61 | 0.2276 |  | 0.8168 |  | 0.1749 |
| HUS1 | 15 | 0.8396 |  | 0.7544 |  | 0.2956 |
| ICAM1 | 20 | 0.0386 |  | 0.1601 |  | 0.4601 |
| IFNB1 | 22 | 0.1254 |  | 0.4984 |  | 0.1506 |
| IFNG | 10 | 0.0050 |  | 0.0511 |  | 0.9136 |
| IFNGR2 | 18 | 0.2856 |  | 0.6325 |  | 0.1531 |
| IL10 | 26 | 0.4261 |  | 0.3818 |  | 0.7753 |
| IL16 | 59 | 0.5673 |  | 0.7810 |  | 0.3309 |
| IL1A | 17 | 0.8089 |  | 0.1916 |  | 0.8554 |
| IL1B | 20 | 0.3377 |  | 0.3940 |  | 0.9620 |
| IL1RN | 51 | 0.6400 |  | 0.8984 |  | 0.3476 |
| IL2 | 10 | 0.2547 |  | 0.8674 |  | 0.7525 |
| IL5 | 13 | 0.9722 |  | 0.4015 |  | 0.6275 |
| IL6 | 33 | 0.6895 |  | 0.9216 |  | 0.0802 |
| IL8 | 21 | 0.1855 |  | 0.8257 |  | 0.8418 |
| IL8RB | 10 | 0.1333 |  | 0.1314 |  | 0.1123 |
| IRF1 | 14 | 0.9273 |  | 0.7087 |  | 0.6606 |
| IRF2 | 114 | 0.3397 |  | 0.6770 |  | 0.5641 |
| IRF3 | 7 | 0.0769 |  | 0.5307 |  | 0.1199 |
| IRF4 | 36 | 0.3089 |  | 0.2564 |  | 0.0890 |
| IRF5 | 12 | 0.9977 |  | 0.9954 |  | 0.8667 |
| IRF7 | 10 | 0.1456 |  | 0.3228 |  | 0.0566 |
| ISGF3G | 11 | 0.5753 |  | 0.8417 |  | 0.6815 |

***Table S2 (continued)***

|  | SNPs | (i)  CIN3/cancer  vs RC |  | (ii)  CIN3/cancer  vs HPV persistence |  | (iii)  HPV persistence  vs RC |
| --- | --- | --- | --- | --- | --- | --- |
| Gene | (#) | p |  | p |  | p |
| ITGA2 | 48 | 0.6503 |  | 0.6341 |  | 0.1146 |
| ITGA4 | 56 | 0.0362 |  | 0.0900 |  | 0.3553 |
| ITGA6 | 87 | 0.8950 |  | 0.4678 |  | 0.0461 |
| ITGA7 | 23 | 0.9855 |  | 0.0384 |  | 0.1073 |
| ITGB1 | 25 | 0.7660 |  | 0.5009 |  | 0.8517 |
| ITGB3 | 36 | 0.8619 |  | 0.1390 |  | 0.4560 |
| ITGB4 | 23 | 0.2419 |  | 0.9359 |  | 0.0650 |
| JAK3 | 29 | 0.6292 |  | 0.2050 |  | 0.3777 |
| LAMA4 | 105 | 0.1431 |  | 0.0902 |  | 0.6478 |
| LIG1 | 36 | 0.7216 |  | 0.9475 |  | 0.5179 |
| LIG3 | 27 | 0.7362 |  | 0.0454 |  | 0.1147 |
| LIG4 | 25 | 0.8954 |  | 0.5191 |  | 0.5779 |
| LTA | 21 | 0.3405 |  | 0.6049 |  | 0.6722 |
| LTF | 20 | 0.3847 |  | 0.2941 |  | 0.3976 |
| MAD2L2 | 13 | 0.5585 |  | 0.3562 |  | 0.1541 |
| MBD4 | 19 | 0.4391 |  | 0.6431 |  | 0.6024 |
| MBL2 | 42 | 0.1602 |  | 0.9814 |  | 0.9829 |
| MGMT | 74 | 0.8051 |  | 0.2203 |  | 0.2294 |
| MLH1 | 28 | 0.2989 |  | 0.3665 |  | 0.4576 |
| MLH3 | 12 | 0.9142 |  | 0.1670 |  | 0.2736 |
| MMS19L | 19 | 0.0340 |  | 0.2986 |  | 0.8535 |
| MNAT1 | 38 | 0.5130 |  | 0.6978 |  | 0.9088 |
| MPG | 15 | 0.6885 |  | 0.8461 |  | 0.6473 |
| MRE11A | 32 | 0.6746 |  | 0.3839 |  | 0.7874 |
| MSH2 | 33 | 0.6887 |  | 0.2594 |  | 0.2197 |
| MSH3 | 44 | 0.4391 |  | 0.4898 |  | 0.7780 |
| MSH4 | 30 | 0.5329 |  | 0.0668 |  | 0.0344 |
| MSH5 | 21 | 0.5298 |  | 0.1544 |  | 0.4620 |
| MSH6 | 24 | 0.8841 |  | 0.3372 |  | 0.1235 |
| MT1G | 18 | 0.2902 |  | 0.3673 |  | 0.1354 |
| MUS81 | 10 | 0.7956 |  | 0.3138 |  | 0.1766 |
| MUTYH | 11 | 0.6512 |  | 0.3649 |  | 0.1328 |
| MYC | 26 | 0.8263 |  | 0.3997 |  | 0.2834 |
| MYD88 | 9 | 0.9720 |  | 0.3714 |  | 0.1729 |
| NBN | 21 | 0.9723 |  | 0.9479 |  | 0.9898 |
| NEIL1 | 4 | 0.7678 |  | 0.8440 |  | 0.3770 |
| NEIL2 | 41 | 0.7735 |  | 0.0042 |  | 0.0079 |
| NEIL3 | 42 | 0.1291 |  | 0.3950 |  | 0.3580 |
| NFKB1 | 40 | 0.5803 |  | 0.7216 |  | 0.2173 |
| NFKB2 | 9 | 0.5797 |  | 0.6834 |  | 0.4436 |
| NOL4 | 97 | 0.5904 |  | 0.5623 |  | 0.1767 |
| NOL7 | 7 | 0.1907 |  | 0.3447 |  | 0.6298 |

***Table S2 (continued)***

|  | SNPs | (i)  CIN3/cancer  vs RC |  | (ii)  CIN3/cancer  vs HPV persistence |  | (iii)  HPV persistence  vs RC |
| --- | --- | --- | --- | --- | --- | --- |
| Gene | (#) | p |  | p |  | p |
| NOS3 | 23 | 0.9624 |  | 0.3623 |  | 0.3709 |
| NOTCH1 | 32 | 0.2982 |  | 0.1622 |  | 0.1686 |
| NOTCH2 | 19 | 0.0841 |  | 0.2988 |  | 0.4995 |
| NTHL1 | 23 | 0.7727 |  | 0.3654 |  | 0.4416 |
| NUDT1 | 15 | 0.4328 |  | 0.9892 |  | 0.7836 |
| OAS1 | 22 | 0.0110 |  | 0.4415 |  | 0.0472 |
| OAS2 | 51 | 0.0134 |  | 0.2968 |  | 0.1041 |
| OAS3 | 36 | 0.0028 |  | 0.2537 |  | 0.1354 |
| OGG1 | 14 | 0.4616 |  | 0.8164 |  | 0.2579 |
| PARP1 | 22 | 0.2200 |  | 0.1308 |  | 0.2155 |
| PARP2 | 28 | 0.1938 |  | 0.1060 |  | 0.0795 |
| PCNA | 8 | 0.1147 |  | 0.2964 |  | 0.1535 |
| PMS1 | 18 | 0.7941 |  | 0.3233 |  | 0.7806 |
| PMS2 | 24 | 0.6801 |  | 0.0851 |  | 0.3749 |
| PMS2L3 | 16 | 0.0652 |  | 0.2217 |  | 0.3271 |
| PNKP | 15 | 0.6666 |  | 0.3887 |  | 0.7900 |
| POLB | 15 | 0.7005 |  | 0.5978 |  | 0.0913 |
| POLD1 | 18 | 0.6192 |  | 0.5803 |  | 0.1679 |
| POLE | 29 | 0.6278 |  | 0.2819 |  | 0.9211 |
| POLG | 33 | 0.6837 |  | 0.6238 |  | 0.4070 |
| POLH | 16 | 0.4626 |  | 0.5221 |  | 0.7517 |
| POLI | 9 | 0.8176 |  | 0.5672 |  | 0.4225 |
| POLK | 23 | 0.1437 |  | 0.7766 |  | 0.6591 |
| POLL | 17 | 0.3715 |  | 0.3504 |  | 0.5110 |
| POLM | 14 | 0.0460 |  | 0.9575 |  | 0.3740 |
| POLN | 35 | 0.0146 |  | 0.2141 |  | 0.3286 |
| POLQ | 31 | 0.4640 |  | 0.0756 |  | 0.1474 |
| PRDM1 | 29 | 0.5981 |  | 0.4077 |  | 0.7812 |
| PRKDC | 16 | 0.0634 |  | 0.2095 |  | 0.7011 |
| PRKRIR | 17 | 0.7964 |  | 0.4060 |  | 0.3324 |
| PTEN | 18 | 0.1366 |  | 0.5947 |  | 0.5631 |
| RAD1 | 14 | 0.4105 |  | 0.4344 |  | 0.3228 |
| RAD17 | 10 | 0.5376 |  | 0.3532 |  | 0.5088 |
| RAD18 | 14 | 0.3191 |  | 0.6229 |  | 0.4783 |
| RAD23A | 7 | 0.1986 |  | 0.6651 |  | 0.5519 |
| RAD23B | 34 | 0.5547 |  | 0.4015 |  | 0.5457 |
| RAD50 | 24 | 0.9729 |  | 0.8664 |  | 0.5213 |
| RAD51 | 11 | 0.5383 |  | 0.7243 |  | 0.6821 |
| RAD51C | 8 | 0.6867 |  | 0.5399 |  | 0.4587 |
| RAD51L3 | 21 | 0.5469 |  | 0.1724 |  | 0.6678 |
| RAD52 | 32 | 0.4271 |  | 0.7543 |  | 0.3517 |

***Table S2 (continued)***

|  | SNPs | (i)  CIN3/cancer  vs RC |  | (ii)  CIN3/cancer  vs HPV persistence |  | (iii)  HPV persistence  vs RC |
| --- | --- | --- | --- | --- | --- | --- |
| Gene | (#) | p |  | p |  | p |
| RAD54B | 29 | 0.9277 |  | 0.1326 |  | 0.4582 |
| RAD54L | 14 | 0.5414 |  | 0.7352 |  | 0.6038 |
| RAD9A | 9 | 0.6691 |  | 0.7228 |  | 0.2368 |
| RARB | 114 | 0.1319 |  | 0.3848 |  | 0.7453 |
| RB1 | 44 | 0.8943 |  | 0.4014 |  | 0.5123 |
| RDM1 | 16 | 0.0357 |  | 0.9047 |  | 0.5259 |
| RECQL | 30 | 0.7510 |  | 0.2543 |  | 0.5465 |
| RECQL4 | 7 | 0.3381 |  | 0.9025 |  | 0.1865 |
| RECQL5 | 6 | 0.2387 |  | 0.8651 |  | 0.1371 |
| REV1 | 16 | 0.4578 |  | 0.4331 |  | 0.4090 |
| REV3L | 24 | 0.3119 |  | 0.4046 |  | 0.1394 |
| RNASEL | 26 | 0.5798 |  | 0.3912 |  | 0.4505 |
| RPA1 | 39 | 0.3754 |  | 0.6121 |  | 0.1518 |
| RPA2 | 17 | 0.2781 |  | 0.7580 |  | 0.2143 |
| RPA3 | 37 | 0.2142 |  | 0.1144 |  | 0.0513 |
| RPA4 | 8 | 0.2060 |  | 0.2962 |  | 0.0261 |
| RRAD | 10 | 0.0661 |  | 0.1248 |  | 0.5026 |
| RRM2B | 21 | 0.2192 |  | 0.8648 |  | 0.2898 |
| SDC1 | 29 | 0.6432 |  | 0.3087 |  | 0.7570 |
| SDC2 | 122 | 0.9407 |  | 0.9929 |  | 0.8148 |
| SDC3 | 41 | 0.2802 |  | 0.2018 |  | 0.2831 |
| SDC4 | 38 | 0.2981 |  | 0.3072 |  | 0.6049 |
| SFRP1 | 49 | 0.1558 |  | 0.0567 |  | 0.7880 |
| SHFM1 | 17 | 0.7400 |  | 0.6213 |  | 0.6648 |
| SMUG1 | 16 | 0.0561 |  | 0.5862 |  | 0.5651 |
| SOCS2 | 17 | 0.5463 |  | 0.2146 |  | 0.0262 |
| SOCS3 | 6 | 0.2990 |  | 0.8149 |  | 0.7831 |
| SPARC | 48 | 0.5592 |  | 0.9340 |  | 0.7360 |
| SPO11 | 14 | 0.0927 |  | 0.7561 |  | 0.1143 |
| SULF1 | 77 | 0.0028 |  | 0.6935 |  | 0.0056 |
| SULF2 | 80 | 0.9181 |  | 0.9976 |  | 0.6347 |
| TADA3L | 14 | 0.3007 |  | 0.4884 |  | 0.5959 |
| TAP2 | 3 | 0.3339 |  | 0.7690 |  | 0.8806 |
| TDG | 34 | 0.0772 |  | 0.7409 |  | 0.3412 |
| TDP1 | 28 | 0.2734 |  | 0.8087 |  | 0.0656 |
| TERC | 7 | 0.8795 |  | 0.8455 |  | 0.5681 |
| TERF1 | 21 | 0.3592 |  | 0.5953 |  | 0.2175 |
| TERF2 | 9 | 0.4152 |  | 0.2233 |  | 0.2409 |
| TERT | 14 | 0.3113 |  | 0.5915 |  | 0.8406 |
| TGFBR1 | 19 | 0.7720 |  | 0.9621 |  | 0.9882 |
| TICAM1 | 18 | 0.1273 |  | 0.8688 |  | 0.7260 |

***Table S2 (continued)***

|  | SNPs | (i)  CIN3/cancer  vs RC |  | (ii)  CIN3/cancer  vs HPV persistence |  | (iii)  HPV persistence  vs RC |
| --- | --- | --- | --- | --- | --- | --- |
| Gene | (#) | p |  | p |  | p |
| TLR2 | 18 | 0.2937 |  | 0.7114 |  | 0.2187 |
| TLR3 | 28 | 0.2628 |  | 0.5880 |  | 0.5658 |
| TLR4 | 41 | 0.9358 |  | 0.6177 |  | 0.9609 |
| TLR6 | 15 | 0.0824 |  | 0.8560 |  | 0.0817 |
| TLR7 | 50 | 0.8161 |  | 0.7807 |  | 0.2829 |
| TLR8 | 40 | 0.4327 |  | 0.5693 |  | 0.5386 |
| TLR9 | 12 | 0.4707 |  | 0.8791 |  | 0.4532 |
| TMC6 | 26 | 0.0082 |  | 0.1357 |  | 0.0028 |
| TMC8 | 22 | 0.0076 |  | 0.0309 |  | 0.8457 |
| TNF | 23 | 0.3733 |  | 0.0287 |  | 0.8114 |
| TNFRSF10C | 19 | 0.7261 |  | 0.6346 |  | 0.8933 |
| TNFRSF14 | 14 | 0.7343 |  | 0.9548 |  | 0.9927 |
| TNFRSF1A | 17 | 0.4054 |  | 0.3386 |  | 0.2280 |
| TNFSF10 | 37 | 0.0297 |  | 0.6851 |  | 0.4845 |
| TNFSF11 | 40 | 0.1062 |  | 0.7991 |  | 0.8254 |
| TOPBP1 | 22 | 0.6715 |  | 0.0306 |  | 0.1138 |
| TP53 | 10 | 0.3493 |  | 0.5757 |  | 0.2633 |
| TP73 | 45 | 0.0769 |  | 0.2948 |  | 0.1850 |
| TRADD | 9 | 0.7447 |  | 0.2240 |  | 0.2643 |
| TREX1 | 13 | 0.4349 |  | 0.8010 |  | 0.5655 |
| TREX2 | 13 | 0.0653 |  | 0.4443 |  | 0.2864 |
| TWIST1 | 13 | 0.6941 |  | 0.3536 |  | 0.4315 |
| UBE2A | 10 | 0.5083 |  | 0.6211 |  | 0.8878 |
| UBE2B | 9 | 0.3818 |  | 0.9644 |  | 0.3518 |
| UBE2N | 15 | 0.7643 |  | 0.3681 |  | 0.8329 |
| UBE2V2 | 6 | 0.5769 |  | 0.6699 |  | 0.2696 |
| UBE3A | 26 | 0.6369 |  | 0.5304 |  | 0.7971 |
| UNG | 26 | 0.0387 |  | 0.3235 |  | 0.4801 |
| VCAM1 | 38 | 0.1463 |  | 0.8233 |  | 0.3011 |
| WRN | 27 | 0.5861 |  | 0.0440 |  | 0.0370 |
| XAB2 | 16 | 0.0631 |  | 0.0925 |  | 0.2011 |
| XPA | 20 | 0.0883 |  | 0.3754 |  | 0.1943 |
| XPC | 45 | 0.2189 |  | 0.5295 |  | 0.7922 |
| XRCC1 | 18 | 0.2337 |  | 0.7603 |  | 0.1519 |
| XRCC2 | 21 | 0.0768 |  | 0.5517 |  | 0.0211 |
| XRCC3 | 26 | 0.1669 |  | 0.3789 |  | 0.4177 |
| XRCC4 | 82 | 0.3071 |  | 0.1765 |  | 0.4913 |
| XRCC5 | 56 | 0.6663 |  | 0.8362 |  | 0.2600 |
| XRCC6 | 15 | 0.1444 |  | 0.8732 |  | 0.0128 |
| XYLT2 | 10 | 0.1850 |  | 0.1101 |  | 0.1127 |
