# Supplementary material for: Common Genetic Variants and Risk for HPV Persistence and Progression to Cervical Cancer
Source: PLoS One. 2010 Jan 13;5(1):e8667. doi: 10.1371/journal.pone.0008667 (PMC2801608; doi:10.1371/journal.pone.0008667)
Supplement: Table S3 — (0.11 MB DOC) [file pone.0008667.s003.doc]

**Table S3**. Odds ratios and 95% confidence intervals for top-ranked SNPs for associations with (i) cervical precancer/cancer, (ii) progression to cervical precancer/cancer, and (iii) HPV persistence, adjusted for age.

| **Gene** | **SNP** | **Genotype** | **Random**  **control** | **HPV**  **persistence** | **CIN3/**  **cancer** | **CIN3/cancer**  **vs RC** | **CIN3/cancer vs**  **HPV persistence** | **HPV persistence**  **vs RC** |
| --- | --- | --- | --- | --- | --- | --- | --- | --- |
|  |  |  | **N (%)** | **N (%)** | **N (%)** | **OR (95% CI)** | **OR (95% CI)** | **OR (95% CI)** |
|  |  |  |  |  |  |  |  |  |
| *DMC1* | RS5757133 | CC  CT  TT | 254 (61)  144 (34)  19 (5) | 233 (66)  106 (30)  12 (3) | 284 (71)  105 (26)  9 (2) | 1.00 (ref)  0.65 (0.48-0.88)  0.39 (0.17-0.88)  p-trend=0.0007 | 1.00 (ref)  0.81 (0.59-1.13)  0.69 (0.28-1.69)  p-trend=0.1590 | 1.00 (ref)  0.80 (0.59-1.09)  0.71 (0.33-1.49)  p-trend=0.1114 |
|  |  |  |  |  |  |  |  |  |
| *DUT* | RS3784621 | TT  CT  CC | 191 (45)  184 (43)  48 (11) | 133 (37)  171 (48)  52 (15) | 139 (34)  203 (49)  70 (17) | 1.0 (ref)  1.57 (1.16-2.12)  1.97 (1.28-3.03)  p-trend=0.0004 | 1.0 (ref)  1.19 (0.86-1.64)  1.39 (0.89-2.16)  p-trend=0.1280 | 1.0 (ref)  1.33 (0.98-1.81)  1.54 (0.98-2.42)  p-trend=0.0262 |
|  |  |  |  |  |  |  |  |  |
| *GTF2H4* | RS2894054 | GG  AG  AA | 313 (74)  103 (24)  9 (2) | 299 (84)  56 (16)  1 (<1) | 349 (84)  62 (15)  3 (1) | 1.0 (ref)  0.53 (0.37-0.75)  0.33 (0.09-1.23)  p-trend=0.0001 | 1.0 (ref)  0.92 (0.61-1.37)  2.25 (0.22-23.02)  p-trend=0.8742 | 1.0 (ref)  0.57 (0.40-0.82)  0.11 (0.01-0.91)  p-trend=0.0002 |
|  |  |  |  |  |  |  |  |  |
| *GTF2H4* | RS6926723 | GG  AG  AA | 318 (75)  100 (24)  7 (25) | 296 (83)  58 (16)  2 (1) | 352 (85)  59 (14)  4 (1) | 1.0 (ref)  0.52 (0.36-0.75)  0.52 (0.15-1.82)  p-trend=0.0004 | 1.0 (ref)  0.84 (0.56-1.27)  1.98 (0.35-11.30)  p-trend=0.6840 | 1.0 (ref)  0.62 (0.44-0.90)  0.30 (0.06-1.44)  p-trend=0.0034 |
|  |  |  |  |  |  |  |  |  |
| *IFNG* | RS11177074 | TT  CT  CC | 387 (91)  37 (9)  1 (<1) | 318 (89)  37 (10)  1 (<1) | 340 (82)  72 (17)  3 (1) | 1.00 (REF)  2.15 (1.40-3.30)  3.12 (0.31-31.2)  p-trend=0.0003 | 1.00 (REF)  1.78 (1.15-2.75)  1.80 (0.19-17.50)  p-trend=0.0095 | 1.00 (REF)  1.23 (0.76-1.99)  1.23 (0.08-19.79)  p-trend=0.4011 |
| *OAS1* | RS12307655 | TT  CT  CC | 376 (89)  46 (11)  0 (0) | 293 (82)  59 (17)  4 (1) | 336 (81)  67 (16)  10 (2) | 1.00 (REF)  1.58 (1.05-2.37)  -  p-trend=0.0005 | 1.00 (REF)  1.08 (0.73-1.60)  2.28 (0.69-7.48)  p-trend=0.2790 | 1.00 (REF)  1.64 (1.08-2.48)  1925317 (0.00-I)  p-trend=0.0034 |
|  |  |  |  |  |  |  |  |  |
| *OAS2* | RS718802 | CC  AC  AA | 362 (85)  61 (14)  2 (1) | 276 (78)  76 (21)  4 (!) | 311 (75)  94 (23)  10 (2) | 1.00 (REF)  1.79 (1.25-2.57)  5.67 (1.22-26.4)  p-trend=0.0001 | 1.00 (REF)  1.17 (0.82-1.66)  2.10 (0.64-6.93)  p-trend=0.1844 | 1.00 (REF)  1.63 (1.12-2.36)  2.67 (0.48-14.70)  p-trend=0.0057 |
|  |  |  |  |  |  |  |  |  |
| *OAS3* | RS12302655 | GG  AG  AA | 369 (87)  56 (13)  0 (0) | 283 (79)  68 (19)  5 (1) | 321 (77)  83 (20)  11 (3) | 1.00 (REF)  1.69 (1.16-2.45)  -  p-trend=0.00008 | 1.00 (REF)  1.16 (0.80-1.67)  1.99 (0.67-5.90)  p-trend=0.1909 | 1.00 (REF)  1.57 (1.07-2.32)  1941546 (0.00-I)  p-trend=0.0025 |
|  |  |  |  |  |  |  |  |  |
| *POLN* | RS17132382 | CC  CT  TT | 283 (69)  113 (27)  16 (4) | 229 (66)  109 (31)  11 (3) | 228 (57)  148 (37)  24 (6) | 1.00 (REF)  1.59 (1.17-2.16)  1.93 (0.99-3.77)  p-trend=0.001 | 1.00 (REF)  1.35 (0.99-1.86)  2.16 (1.02-4.59)  p-trend=0.0103 | 1.00 (REF)  1.20 (0.87-1.64)  0.86 (0.39-1.89)  p-trend=0.5304 |
|  |  |  |  |  |  |  |  |  |
| *SULF1* | RS4737999 | GG  AG  AA | 229 (54)  163 (38)  33 (8) | 237 (67)  99 (28)  20 (6) | 270 (65)  130 (31)  15 (4) | 1.0 (ref)  0.66 (0.49-0.88)  0.36 (0.19-0.68)  p-trend=0.0001 | 1.0 (ref)  1.16 (0.84-1.60)  0.68 (0.33-1.37)  p-trend=0.9556 | 1.0 (ref)  0.59 (0.43-0.81)  0.59 (0.33-1.06)  p-trend=0.0011 |
|  |  |  |  |  |  |  |  |  |

***Table S3 (continued)***

| **Gene** | **SNP** | **Genotype** | **Random**  **control** | **HPV**  **persistence** | **CIN3/**  **cancer** | **CIN3/cancer**  **vs RC** | **CIN3/cancer vs**  **HPV persistence** | **HPV persistence**  **vs RC** |
| --- | --- | --- | --- | --- | --- | --- | --- | --- |
|  |  |  | **N (%)** | **N (%)** | **N (%)** | **OR (95% CI)** | **OR (95% CI)** | **OR (95% CI)** |
|  |  |  |  |  |  |  |  |  |
| *SULF1* | RS4284050 | CC  AC  AA | 159 (37)  213 (50)  53 (12) | 169 (48)  148 (42)  39 (11) | 205 (49)  171 (41)  39 (9) | 1. (ref)   0.62 (0.46-0.83)  0.55 (0.34-0.87)  p-trend=0.0007 | 1. (ref)   0.94 (0.69-1.27)  0.86 (0.52-1.41)  p-trend=0.5160 | 1.0 (ref)  0.66 (0.49-0.89)  0.69 (0.44-1.11)  p-trend=0.0181 |
|  |  |  |  |  |  |  |  |  |
| *SULF1* | RS10108002 | CC  CT  TT | 224 (53)  178 (42)  22 (5) | 232 (65)  109 (31)  15 (4) | 263 (63)  140 (34)  12 (3) | 1.0 (ref)  0.65 (0.49-0.87)  0.47 (0.23-0.99)  p-trend=0.001 | 1.0 (ref)  1.10 (0.80-1.50)  0.80 (0.36-1.79)  p-trend=0.8918 | 1.0 (ref)  0.60 (0.44-0.81)  0.66 (0.33-1.30)  p-trend=0.0020 |
|  |  |  |  |  |  |  |  |  |
| *TMC8,*  *TMC6* | RS9893818 | CC  AC  AA | 391 (95)  21 (5)  0 (0) | 328 (95)  19 (5)  0 (0) | 295 (87)  44 (13)  0 (0) | 1.00 (REF)  2.76 (1.59-4.77)  -  p-trend=0.0003 | 1.00 (REF)  2.47 (1.40-4.36)  (-)  p-trend=0.0019 | 1.00 (REF)  1.07 (0.56-2.02)  (-)  p-trend=0.8434 |
|  |  |  |  |  |  |  |  |  |
