# Supplementary material for: Common Genetic Variants and Risk for HPV Persistence and Progression to Cervical Cancer
Source: PLoS One. 2010 Jan 13;5(1):e8667. doi: 10.1371/journal.pone.0008667 (PMC2801608; doi:10.1371/journal.pone.0008667)
Supplement: Table S5 — (0.09 MB DOC) [file pone.0008667.s005.doc]

**Table S5**. SNP and haplotype results for selected haplotype blocks (as defined by Haploview 3.32) in *DUT*, *GTF2H4*, and *SULF1.*

| **Gene** | **SNP/haplotype** | **Random control** | **HPV persistence** | **CIN3/cancer** | **CIN3/cancer**  **vs RC** |
| --- | --- | --- | --- | --- | --- |
|  |  | **N (%)** | **N (%)** | **N (%)** | **OR (95% CI)** |
| ***DUT*** | **RS3784621** |  |  |  |  |
|  | **TT**  **CT**  **CC** | 191 (45)  184 (43)  48 (11) | 133 (37)  171 (48)  52 (15) | 139 (34)  203 (49)  70 (17) | 1. (ref)   1.57 (1.16-2.12)  1.97 (1.28-3.03)  p-trend=0.0004 |
|  | RS3784619  RS11857732  RS7175137  RS16960782  **RS3784621**  RS11637235  RS12441867  RS13379705 |  |  |  |  |
|  | A C T A **C** C T T | 13% |  |  | 1.87 (1.37-2.56) |
|  | A C T A T C C T | 24% |  |  | 1.15 (0.90-1.47) |
|  | G C T A **C** C T C | 20% |  |  | 1.25 (0.97-1.63) |
|  | A C T A T T C T | 37% |  |  | 1.0 (ref) |
|  |  |  |  |  |  |
| ***GTF2H4*** | **RS2894054**  **GG**  **AG**  **AA** | 313 (74)  103 (24)  9 (2) | 299 (84)  56 (16)  1 (<1) | 349 (84)  62 (15)  3 (1) | 1.0 (ref)  0.53 (0.37-0.75)  0.33 (0.09-1.23)  p-trend=0.0001 |
|  | **RS6926723**  **GG**  **AG**  **AA** | 318 (75)  100 (24)  7 (25) | 296 (83)  58 (16)  2 (1) | 352 (85)  59 (14)  4 (1) | 1.0 (ref)  0.52 (0.36-0.75)  0.52 (0.15-1.82)  p-trend=0.0004 |
|  | \RS1049628  RS9468843  RS9378150  **RS2894054**  RS3130780  RS1264308  RS3218819  RS3218831  **RS6926723**  RS2285319  RS2249464  RS2074511 |  |  |  |  |
|  | C T A **A**  G C T C **A**  C T T | 6% |  |  | 0.46 (0.29-0.72) |
|  | C T A G G C T C G C C T | 20% |  |  | 0.88 (0.67-1.17) |
|  | C T A G T C T C G C T T | 18% |  |  | 1.01 (0.76-1.35) |
|  | T C A G G C T C G T C C | 31% |  |  | 1.0 (ref) |

*Table S5* (continued)

| *SULF1* | **RS7845415**  **AA**  **AG**  **GG** | 171 (40)  202 (47)  52 (12) | 111 (31)  171 (48)  74 (21) | 136 (33)  209 (50)  70 (17) | 1.0 (ref)  1.27 (0.94-1.72)  1.66 (1.08-2.56)  p-trend=0.02 |
| --- | --- | --- | --- | --- | --- |
|  | RS920529  **RS7845415**  RS17646998  RS723290  RS10108002 |  |  |  |  |
|  | A **A** T C T | 21% |  |  | 0.56 (0.45-0.77) |
|  | T **A** C C C | 6% |  |  | 0.90 (0.58-1.39) |
|  | T **A** C T C | 23% |  |  | 0.84 (0.65-1.09) |
|  | A G C C C | 38% |  |  | 1.0 (ref) |
|  |  |  |  |  |  |
|  | **RS17646998**  **CC**  **CT**  **TT** | 192 (45)  193 (46)  39 (9) | 205 (58)  126 (35)  25 (7) | 225 (54)  163 (39)  27 (7) | 1.0 (ref)  0.72 (0.54-0.96)  0.58 (0.34-1.00)  p-trend=0.008 |
|  | **RS10108002**  **CC**  **CT**  **TT** | 224 (53)  178 (42)  22 (5) | 232 (65)  109 (31)  15 (4) | 263 (63)  140 (34)  12 (3) | 1.0 (ref)  0.65 (0.49-0.87)  0.47 (0.23-0.99)  p-trend=0.001 |
|  | **RS4737999**  **GG**  **AG**  **AA** | 229 (54)  163 (38)  33 (8) | 237 (67)  99 (28)  20 (6) | 270 (65)  130 (31)  15 (4) | 1.0 (ref)  0.66 (0.49-0.88)  0.36 (0.19-0.68)  p-trend=0.0001 |
|  | **RS4284050**  **CC**  **AC**  **AA** | 159 (37)  213 (50)  53 (12) | 169 (48)  148 (42)  39 (11) | 205 (49)  171 (41)  39 (9) | 1.0 (ref)  0.62 (0.46-0.83)  0.55 (0.34-0.87)  p-trend=0.0007 |
|  | RS920529  RS7845415  **RS17646998**  RS723290  **RS10108002**  RS13273088  RS12545167  RS17720343  RS7846303  RS4737998  RS6472468  **RS4737999**  RS17664415  RS1866898  RS1866897  RS6472471  **RS4284050**  RS12543013  RS4738009 |  |  |  |  |
|  | A A  **T** C **T** A G G A G A **A** C A C A **A** A G | 13% |  |  | 0.51 (0.36-0.74) |
|  | A G C C C G A G A G G G C T C C C A G | 6% |  |  | 1.10 (0.69-1.76) |
|  | T A C T C A G G T T G G C T A A C G G | 10% |  |  | 0.84 (0.57-1.23) |
|  | A G C C C G A G A T G G C T A A C G G | 20% |  |  | 1.0 (ref) |
